# Risk of Cancer in Patients with Inflammatory Bowel Diseases and Keys for Patient Management

**DOI:** 10.3390/cancers15030871

**Published:** 2023-01-31

**Authors:** Viviana Laredo, Sandra García-Mateo, Samuel J. Martínez-Domínguez, Julia López de la Cruz, Carla J. Gargallo-Puyuelo, Fernando Gomollón

**Affiliations:** 1 Department of Gastroenterology, Lozano Blesa University Hospital, 50009 Zaragoza, Spain; 2 Aragón Health Research Institute (IIS Aragón), 50009 Zaragoza, Spain; 3 School of Medicine, University of Zaragoza, 50009 Zaragoza, Spain; 4 CIBER for Liver and Digestive Diseases (CIBERehd), 28029 Madrid, Spain

**Keywords:** Inflammatory Bowel Disease, cancer, immunosuppressive drugs, biologic therapy, tofacitinib, treatment decisions

## Abstract

**Simple Summary:**

Inflammatory Bowel Diseases (IBDs) are increasingly prevalent pathologies due to the rise in incidence in some geographic areas and the improvement in prognosis with the development of new therapeutic targets. The increasing age of the IBD population is associated with greater comorbidity, including intestinal and extra-intestinal cancer. The current or previous presence of cancer makes the treatment of both IBD and cancer challenging. In this review, we summarize the evidence on IBD cancer risk related to chronic inflammation and immunosuppressive therapy, as well as a general approach to the management of patients with IBD and cancer.

**Abstract:**

Chronic inflammation in patients with Inflammatory Bowel Disease (IBD) leads to an increased risk of colorectal cancer, small bowel cancer, intestinal lymphoma and cholangiocarcinoma. However, treatments for IBD have also been associated with an increased risk of neoplasms. Patients receiving Thiopurines (TPs) have an increased risk of hematologic malignancies, non-melanoma skin cancer, urinary tract neoplasms and cervical cancer. Anti-TNFs have been associated with a higher risk of neoplasms, mainly lymphomas and melanomas; however, the data are controversial, and some recent studies do not confirm the association. Nevertheless, other biologic agents, such as ustekinumab and vedolizumab, have not shown an increased risk of any neoplasm to date. The risk of malignancies with tofacitinib exists, but its magnitude and relationship with previous treatment with TPs is not defined, so more studies from daily clinical practice are needed. Although biologic therapy seems to be safe for patients with current cancer or a prior history of cancer, as has been demonstrated in other chronic inflammatory conditions, prospective studies in this specific population are needed. Until that time, it is crucial to manage such conditions via the combined clinical expertise of the gastroenterologist and oncologist.

## 1. Introduction

Inflammatory Bowel Diseases (IBDs) are chronic inflammatory pathologies that mainly involve the gastrointestinal tract, alternating relapses of inflammation and periods of remission. IBDs include ulcerative colitis (UC), Crohn’s disease (CD) and indeterminate colitis (IC) [1]. The global burden of IBD has increased due to higher incidence, longer patient survival and the improvement of diagnostic techniques [1,2,3,4,5].

Age is the most important risk factor for cancer, and the trend of the IBD population is increasing age, so exposure to malignancies is rising. In addition, chronic inflammation and some treatments for IBD are also risk factors for neoplastic changes. The release of proinflammatory mediators during IBD relapses leads to carcinogenesis by promoting oxidative stress that favors DNA mutagenesis, antiapoptotic pathways and migration, angiogenesis and invasion phenomena [6].

Indeed, treatment of current neoplasms in IBD patients, as well as the treatment of IBD in patients with prior malignancies, has become a challenge with limited evidence-based data available, since patients with an active or recent history of cancer or those who develop cancer while being treated with newer therapies are usually excluded from randomized controlled trials. Consequently, it is essential to consider primary and secondary prevention measures for neoplasms during daily clinical practice in IBD units [7].

Therefore, this article aims to review the risk of cancer related to chronic inflammation in IBD and immunosuppressive or biologic treatments and, in addition, to propose a general therapeutic strategy for patients suffering from IBD and neoplasia.

## 2. Literature Review

The search was conducted around October 2022 in PubMed, Embase, Science-Direct and Medline. We used the following keywords: “Inflammatory Bowel Disease”, “Crohn’s Disease”, “Ulcerative Colitis”, “cancer”, “neoplasms”, “thiopurines”, “biologic agents”, “anti-TNF”, “vedolizumab”, “ustekinumab”, “tofacitinib” and “management”. Additionally, a manual search of the literature search was performed. Most relevant original manuscripts written in English were selected for revision after access to full text versions through open-access licenses or institutional access.

## 3. Chronic Inflammation and Cancer Risk

### 3.1. Colorectal Cancer

#### 3.1.1. Epidemiology and Risk Factors

Colorectal cancer (CRC) is the third cause of cancer worldwide, being especially common in developed countries. Although CRC is a very prevalent condition, mortality is decreasing due to screening programs and the improvement of therapeutic strategies [8].

UC is known as a well-established risk factor for CRC [8,9,10,11], with a risk of 1% by 10 years, 2% by 20 years and 5% by >20 years [12]. Indeed, a 4–10-fold increased incidence compared with sporadic CRC has been reported in patients with UC [13]. Nevertheless, the association between CD and CRC has been more controversial [14,15,16]. However, the CRC incidence in patients with IBD is declining over the years probably due to an improved control of inflammation, maintenance therapy utilization, access to surgery in case of medically resistant disease and surveillance programs implementation [12,15,16].

Several IBD-related risk factors have been described, and their presence guides the most appropriate screening and surveillance strategy. Primary Sclerosing Cholangitis (PSC) and severe inflammatory activity are the most consistent risk factors described [17,18]. The presence of post-inflammatory polyps traduces previous inflammatory severity and can make it difficult to detect adenomas. Although traditionally pseudopolyps have been associated with an increased risk of CRC [19,20], recent data suggest that they are not a risk factor for CRC [21]. In addition, younger age at diagnosis of IBD and longer duration of IBD have been described as risk factors for CRC [14]. Extension of IBD also influences CRC risk, ranging from no increased risk in proctitis to the highest risk for extensive colitis, while left-sided colitis shows an intermediate CRC risk. Other risk factors are family history of first-degree relative with CRC and male gender [22].

#### 3.1.2. Pathogenesis

IBD pathogenesis is not fully established, and it is believed to be a multifactorial process resulting from the combination of genetic susceptibility and environmental factors such as diet, smoking habit or stress [23]. IBD has been related to hygienic conditions, dietary habits and overutilization of antibiotics, suggesting a probable relationship with microbiota [24]. In fact, lower levels of bacteria associated with an anti-inflammatory effect, such as *Faecalibacerium prausnitzii* or *Roseburia* spp., have been detected in IBD. Additionally, mucosal damage allows pathogenic bacteria such as *Proteobacteria* to penetrate and promote inflammatory processes [25].

Tumorigenesis in patients with IBD follows the inflammation–dysplasia carcinoma sequence instead of adenoma–carcinoma sequence described for sporadic CRC. Molecular alterations in sporadic CRC appear in a different order and frequency in IBD-related CRC. Whereas p53 mutation occurs earlier in IBD-related CRC, mutations in APC gatekeeper gene occur later, just prior to carcinoma [26]. Three theories about bacterial involvement in IBD-related CRC have been proposed: the alpha-bug hypothesis, driver–passenger hypothesis and common-ground hypothesis. The first hypothesis affirms that a single bacterium causes carcinogenesis, while the second believes that, after the effect of this first bacterium, other opportunistic bacteria contribute. The common-ground hypothesis states that increased bowel permeability allows bacterial invasion of submucosal tissue, leading chronic inflammation and cancer [13].

#### 3.1.3. Screening and Surveillance

The goal of screening programs is an early detection of CRC cancer, based on the early detection of dysplasia and, for that purpose, three screening systems have been developed [27]. Traditionally, dysplasia was investigated in each colonic segment, using quadrantic random biopsies every 10 cm, plus targeted biopsies of any visible lesion [28,29]. Although this system is not strongly recommended by the American College of Gastroenterology (ACG) and the British Society of Gastroenterology (BSG) at this moment [30,31], the European Crohn´s and Colitis Organisation (ECCO) [27] guidelines recommend it when using only white-light scopes. With the improvement of image quality in endoscopy, colonoscopy with dye spray chromoendoscopy using methylene blue or indigo carmine, adding targeted biopsies to areas suspicious of dysplasia, has become the gold-standard screening system, especially recommended in high-risk patients (for example, in previous Low-Grade Dysplasia (LGD)). However, virtual chromoendoscopy with high definition colonoscopes is an adequate alternative in low- and moderate-risk patients [32,33]. As most neoplastic lesions are visible due to technological improvements, the role of random biopsies has been progressively relegated to specific situations. Nevertheless, local factors often influence the screening system used [27,28,34].

Most guidelines recommend starting screening colonoscopy over 8 years following the onset of symptoms for all patients, except for patients with PSC (annual surveillance colonoscopy following diagnosis) and proctitis (no specific surveillance program is needed) [27,30,31]. Ongoing surveillance colonoscopy depends on the presence of high- or intermediate-risk features and varies according to different guidelines, with the recent ones ECCO 2017, ACG 2019 and BSG 2019 recommendations (Table 1). However, available evidence from prospective randomized controlled trials is scarce.

#### 3.1.4. Treatment

An accurate histopathological diagnosis of dysplasia and description of endoscopic features (polypoid lesions, non-polypoid lesions, invisible) is a cornerstone to choose the most appropriate therapeutic strategy [27,28,34]. Parallel to the development of endoscopic techniques, the traditional paradigm of recommending proctocolectomy for all patients with any form of dysplasia has changed.

In the case of finding dysplasia in a colonic segment currently or previously affected by colitis, it is necessary to consider if it comes from a visible lesion, its endoscopic resectability and the presence of multiple foci. Conservative management can be performed, avoiding surgery, when polypoid dysplasia or non-polypoid dysplasia can be completely excised and there is no evidence of non-polypoid or invisible dysplasia elsewhere in the colon [29,30,31].

Due to the higher risk of high-grade dysplasia (HGD) or CRC development at 5 years for non-polypoid dysplasia (65.2%) compared to polypoid dysplasia (6.0%), unresectable non-polypoid dysplasia requires colectomy, regardless of the grade of dysplasia. Nevertheless, unresectable polypoid dysplasia can be managed with partial colonic resection or surveillance in the case of low-grade dysplasia [28]. Surveillance colonoscopy is recommended at 3–6 months before returning to annual surveillance after complete resection of both polypoid and non-polypoid dysplastic lesions [28].

In the case of invisible dysplasia, the first step is to perform a chromo-endoscopy with a high-definition endoscope in a referral center to rule out visible lesions missed on the previous colonoscopy. If no visible lesion is identified, its management depends on the grade of initial dysplasia: in the case of HGD, the patient should be referred to colectomy, while in the case of LGD, an individualized decision between colectomy or surveillance should be taken as 5 years of HGD or if the CRC rate is 21.9% (higher than polypoid lesions but lower than non-polypoid lesions) [35]. Polyps that arise proximal to segments involved are considered to be sporadic adenomas and should be treated accordingly [28].

#### 3.1.5. Primary Prevention

Nowadays, primary prevention does not replace screening and surveillance strategy. The most important strategy for primary prevention is control of inflammation, because this considerably reduces the risk of neoplasia. However, different drugs have been tested for this purpose, such as5-Aminosalicylic Acid (5-ASA), which is weakly recommended by BSG and ECCO, whereas ACG does not recommend it; and thiopurines (TPs), which are not recommended by international guidelines [27,28,34].

Furthermore, 5-ASA has been proposed as a preventive measure for CRC through the downregulation of cyclooxygenase-2 (COX-2) and inhibiting activation of transcription of nuclear factor kB (NF-kB) and phospholipase D. Indeed, several retrospective case-control studies found a significant protective effect of 5-ASA in UC, especially with doses >1.2 g/day. However, it is unknown if this is related to an improved inflammation control instead of being a specific drug effect. There is a lack of randomized prospective studies confirming these findings [36,37].

Evidence regarding the chemoprotective effect of TPs and anti-TNFs is shown later in this review.

### 3.2. Small Bowel Neoplasia

A recently published nationwide cohort study performed in Sweden and Denmark revealed an eight-fold and two-fold increased risk of small bowel adenocarcinoma in CD and UC, respectively [38]. Only patients with extensive UC were at increased risk, while both patients with CD affecting small bowel/ileocecal region or colon had a high risk. Recent diagnosis, ileal involvement and stricturing disease have been reported as risk factors in patients with CD. PSC is also a risk factor in patients with UC [39]. In addition, the risk of neuroendocrine tumors was increased by about two-fold in both UC and CD [40,41].

Contrary to CRC, no optimal strategies for the screening of small bowel cancer are available. Full visualization through ileoscopy may be inadequate, and although findings in cross-sectional imaging can be suggestive of neoplasia, most cases are usually diagnosed during surgery of complications. In the case of long-standing persistent stenosis, surgery should be considered. No drug has demonstrated efficacy in primary prevention [42].

### 3.3. Intestinal Lymphoma

Primary intestinal lymphoma is mainly associated with immunosuppressant agents such as TPs, although chronic intestinal inflammation could also play a role [42,43]. Similar to the general population, B-cell non-Hodgkin´s lymphoma is the most common subtype in patients with IBD (up to 83.9%). Nevertheless, primary intestinal lymphoma is overrepresented in contrast to the general population. Between 4% and 75% of patients have positive Epstein–Barr Virus status, so preventive measures can be intensified in that group [38].

### 3.4. Cholangiocarcinoma

Cholangiocarcinoma is a rare condition with poor prognosis that, in most cases, is not associated with any recognized risk factor [44]. However, a two- to four-fold higher risk of cholangiocarcinoma has been detected in patients with IBD compared to the general population [45]. When comparing UC and CD, a two-fold increased risk for UC has been described. Other risk factors are smoking habit, alcohol, older age at PSC diagnosis and the presence of CRC or dysplasia in UC patients [46].

No specific surveillance recommendations have been made for patients with IBD, so recommendations from liver scientific associations could be taken as reference. Cross-sectional imaging (Magnetic Resonance Imaging or ultrasonography) and CA 19.9 have been suggested for cholangiocarcinoma screening every 6–12 months in PSC patients by American guidelines and some experts; however, ECCO does not support this strategy specifically [46,47,48,49,50,51].

## 4. IBD Existing Therapies and Cancer Risk

At the present, there are many drugs for IBD treatment which module the immune response: immunosuppressants, mainly TPs and methotrexate (MTX); biologic therapies, such as anti-TNFs, anti-integrin and anti-IL12/23; and, recently, small molecules, such as jam-kinase inhibitors and SP1 modulators. The efficacy of these therapies has been variably demonstrated in randomized clinical trials, and they are recommended by clinical guidelines; however, these drugs have also been associated with an increased risk of infections and neoplasms [47,48,49,50,51,52].

### 4.1. Thiopurines

TPs (azathioprine and mercaptopurine) have been associated with an increased risk of hematologic malignancies, non-melanoma skin cancer (NMSC) and urinary-tract neoplasms [53,54], while the association with dysplasia and cervical cancer is controversial [50,55,56,57]. The risk of cancer seems to be especially increased in older patients and in combo-therapies with anti-TNFs [50,58,59]. A Spanish study with the ENEIDA registry including 48,752 IBD patients treated with TPs reported that patients who initiated TPs above 60 years old had an increased risk of neoplasms (1.5% vs. 0.2%; *p* < 0.001), and in 88.5% of patients, it leads to treatment discontinuation [58].

Regarding the *risk of hematologic malignancies,* a French cohort of 19,486 IBD patients reported an increased risk of lymphoproliferative diseases (mainly lymphoma) of five times for patients treated with TPs compared to those never exposed (adjusted HR (aHR), 5.28; 95% CI, 2.01–13.9; *p* = 0.0007) [55]. Furthermore, the risk of neoplasm was higher for those with active treatment (2% vs. 1% vs. 1%; *p* = 0.0016), finding no differences in the adjusted risk between patients with previous treatment and those never exposed (aHR 1.02; 95% CI 0.20–5.11; *p* = 0.9839). In a recent study, the risk of lymphoma in IBD patients treated with TPs was similar to the French cohort (1.04 per 1000 patients-year) [60]. It should be noted that although the risk of lymphoma is increased with TPs, the incidence remains relatively low (0.9–1 per 1000 patients-year), and the risk is reversible after TP withdrawal [55,61].

It also seems to be an association between the risk of lymphoma, Epstein–Barr virus (EBV) infection and TP therapy in IBD patients, probably due to T-cell apoptosis, leading to uncontrolled proliferation of B cells infected by EBV [55,62]. Low TP compliance has also been associated with an increased risk of EBV–lymphoma by an imbalance between cell inhibition and proliferation [63]. Fatal lymphoproliferative disorders have been reported in young male EBV–seronegative IBD patients under TP therapy; however, this risk is low (1 in 10,000) [55]. The risk of lymphoma is higher in CD and with persistent inflammation [64,65]. Hepatosplenic T-cell lymphoma has also been associated with TP monotherapy and combo-therapy, although the risk is low [64].

In addition, the risk of acute myeloid leukemia and myelodysplastic syndrome is also higher in patients with active TP therapy (17.0 per 100,000 patients-years in never exposed, 17.7 in past exposure and 30.4 in active TP therapy < 2 years-and 30.3 ≥ 2 years) [66]. Active TP therapy, but not previous exposure, increases the risk of this neoplasm when compared with no exposure (aHR = 3.05 for active TP < 2 years, *p* = 0.0014; aHR = 2.32 for active TP ≥ 2 years, *p* = 0.0101; aHR = 1.47, *p* = 0.2110 for previous exposure). All patients were diagnosed when they were above 51 years old.

In IBD patients, the risk of *melanoma and NMSC* is increased compared to the general population (IRR = 1.29; 95% CI = 1.09–1.53 and IRR = 1.46; 95% CI = 1.40–1.53), especially in CD [67]. TPs have also been associated with an increased risk of NMSC (OR = 1.85; 95% CI = 1.66–2.05) that persists even when TP therapy is discontinued (5.9 HR and 3.9 HR for active and prior use, respectively) [56,67]. On the contrary, no association has been found between TPs and the risk of melanoma (OR = 1.10; 95% CI = 0.72–1.67) [67]. In fact, in a recent retrospective study, TP therapy was found to increase the risk of basal cell carcinoma (HR = 1.52; 95% CI = 1.10–2.10) and squamous cell carcinoma (HR = 7.81; 95% CI = 4.56–11.05) but not melanoma (HR = 1.51; 95% CI = 0.50–4-54) [68]. However, the risk of NMSC attributable to TPs is difficult to interpret since studies are heterogeneous and many factors involved in the risk of skin cancer are not taken into account (sun exposure, skin phenotype, etc.) [68,69].

Concerning urinary-tract neoplasms, in the CESAME cohort, active TP therapy increased the risk compared with no treatment (aHR 2.82; *p* = 0.04) [50]. Globally, the incidence rate was 0.32/1000 patients-year, and it increases to 0.48/1000 patient-years in active TP therapy and to 9.6/1000 patients-year in men older than 65 years old with active TP therapy. The risk is similar to the general population in those with previous TP exposure. In a recently published Dutch study, the incidence rate of urinary neoplasms under TP therapy was lower than in the CESAME cohort (0.21/1000 patients-year) [60]. Other factors may also modify this risk, such as Human Papilloma Virus (HPV) infection, which has been associated with an increased risk of squamous bladder cancer [70,71].

Regarding the risk of other neoplasms, no association has been found between TPs and cholangiocarcinoma or gallbladder cancer (HR 1.05; 95% CI 0.39–3.82) [72,73]. Concerning gynecologic malignancies, although TPs have been associated with an increased risk of an abnormal pap test [57,74], a recent meta-analysis did not find an increased risk of cervical cancer in IBD patients treated with TPs (HR 0.96, 95% CI 0.60–1.50).

Despite all of the abovementioned, TPs have also been associated with a decreased risk of CRC in IBD patients. In the CESAME cohort, in patients with long-term extensive colitis, TP therapy was a protector factor for high-grade dysplasia and CRC (aHR = 0.28; 95% CI = 0.1–0.9; *p* = 0.03), probably due to a better inflammation control [22]. Similar results were reported from the ENEIDA database, including 831 UC patients, with TP exposure as an independent protective factor for CRC (OR = 0.21; 95% CI = 0.06–0.74; *p* = 0.015) [75]. The protective effect was also found in a recent meta-analyses, including 95,397 patients; however, it was limited to patients with long-term disease (more than 8 years) [76]. On the other hand, a case-control study in the CESAME cohort failed to show a chemoprotective effect of TPs in CRC; however, authors indicate that the study was probably underpowered [77].

### 4.2. Anti-TNF

In observational studies, anti-TNFs have been associated with an increased risk of cancer, mainly NMSC, melanoma and lymphoma, and less probably with its association with other types of neoplasms [59,68]. However, some recent meta-analysis and observational studies do not confirm these findings and suggest that the increased risk of cancer could be limited to anti-TNFs in combo-therapies [46]. In a Denmark registry including 56,146 IBD patients, after adjustment for age, disease duration and TP exposure, there was not an increased risk of cancer associated with anti-TNFs (RR = 1.07; 95% CI = 0.85–1.36) [52]. In the TREAT long-term prospective registry of CD patients, the risk of malignancy was similar between anti-TNFs (infliximab) and other therapies [78]. In a study in the ENEIDA cohort, including 11,011 IBD patients, immunosuppressants and anti-TNFs were not associated with an increased risk of cancer (HR = 0.73; 95% CI = 0.56–0.96 and HR = 0.72; 95% CI = 0.52–1.001, respectively) [69]. In another study including CD patients treated with adalimumab, combo-therapy but not monotherapy was associated with an increased risk of NMSC (RR = 3.46; 95% CI = 1.08–11.06) and other neoplasms (RR = 2.82; 95% CI = 1.07–7.44) [79]. In a recently published systematic review trying to assess the risk of malignancy of anti-TNFs, eleven studies evaluated the association between anti-TNFs and malignancy, showing no increased risk in ten of them [80].

Regarding melanoma, in a case-control study using an administrative database, anti-TNF therapy was associated with an increased risk of melanoma, even after adjustment (OR = 1.88; 95% CI = 1.08–3.29), but not in the case of NMSC (OR = 1.14; 95% CI = 0.95–1.36) [67]. Another study also found no association between infliximab and NMSC [78], but in a retrospective study, anti-TNFs increase the risk of basal cell carcinoma (HR = 1.76; 95% CI = 1.08–2.87) and melanoma (HR = 4.1; 95% CI = 1.32–12.73) but not squamous cell carcinoma (HR = 1.55; 95% CI = 0.48–5.04) [68]. Treatment with combo-therapy for more than one year has been associated with the highest risk of NMSC (OR = 3.89; 95% CI = 2.33–6.46) [67], and it has also been associated with an increased risk of squamous cell carcinoma (HR = 7.83; 95% CI = 3.41–17.98) [68]. Another study also reported an increased risk of NMSC in patients treated with combo-therapy [79].

The risk of lymphoma has also been investigated; however, assessing the risk of anti-TNF monotherapy can be difficult because many patients have been previously treated with TPs [64,81,82]. In a French cohort of 189,289 IBD patients, the incidence rate of this neoplasm was higher in patients treated with anti-TNF monotherapy or combo-therapy when compared with those unexposed (aHR = 2.41, *p* < 0.01; and aHR = 6.11, *p* < 0.01, respectively) [59]. Moreover, combo-therapy significantly increases the risk of lymphoma when compared to anti-TNF or TP monotherapy (aHR = 2.53, *p* < 0.001; and aHR = 2.35, *p* < 0.01, respectively). Despite this increased risk, the absolute risk is low with all therapeutic options (IR = 0.54 per 1000 person-years for TPs, 0.41 for anti-TNFs and 0.95 for combo-therapy). When comparing TP and anti-TNF monotherapy, there were no differences in the lymphoma risk (aHR = 0.93; 95% CI = 0.60–1.44). In a recent systematic review including 261,689 patients, combo-therapy (IRR = 3.71 per 1000 patients-year; *p* ≤ 0.01) and monotherapy (IRR = 1.52 per 1000 patients-year; *p* = 0.023) were also associated with an increased risk of lymphoma compared with no exposure to anti-TNFs or TPs [49] On the contrary, other studies did not find an increased risk of lymphoma in patients treated with anti-TNF monotherapy [83,84].

The most frequent lymphomas developed under immunosuppressive therapy are B-cell, follicular and Hodgkin lymphoma [59,85,86]. In a case-series from the CONFER project, 15 patients with intestinal lymphoma were included, and most of them were men (12/15) and affected by CD (11/15) [43]. In 10 of 15 patients, the location was the IBD affected area, and 9 patients had history of anti-TNF or TP exposure [87].

Contrary to TPs, anti-TNF therapy does not increase the risk of acute myeloid leukemia and myelodysplastic syndrome (aHR = 0.86; *p* = 0.7462) [66].

The evidence of anti-TNFs in CRC chemoprevention is scarce; however, the implication of the TNF factor in the development of this neoplasm suggests the potential protective effect of these drugs [88]. In a U.S. cohort of IBD patients, anti-TNF therapy was found to be a protective factor for the development of CRC in both CD and UC (OR = 0.69, 95% CI = 0.66–0.73, *p* < 0.0001; and OR = 0.78, 95% CI = 0.73–0.83, *p* < 0.0001) [89]. However, in a case-control study including 41,176 patients from a Canadian database, there was no association between anti-TNFs and CRC risk [90], and, in a Danish study, a protective effect of anti-TNFs was not found [91].

### 4.3. Vedolizumab

Vedolizumab (VDZ) is a gut-selective monoclonal antibody against α4β7 integrin that is approved for the treatment of moderately to severely active UC and CD. Although long-term follow-up and real-world evidence are scarce, safety analyses of clinical trials and open-label extension studies have not observed any significant increase in the risk of solid-organ or hematologic malignancies. In the GEMINI long-term safety (LTS) study, no trend was observed between the development of malignancy and age, sex, type of malignancy or duration of VDZ exposure. The most common malignant neoplasm was basal cell carcinoma, with a <1% incidence in UC and CD. The rate of all-site malignancies was 9.8/1000 per year in UC and 8.3 in CD, suggesting that VDZ does not impact the overall rate of malignancies [92].

Therefore, Colombel et al. in a phase 2/3 study did not report an increased risk of malignancy [93]. A retrospective cohort study supported this findings compared with anti-TNFs, and no difference was observed in the incidence of malignancy between VDZ versus TNFα antagonists (IR = 1.28; 95% CI = 0.61–2.45) [94]. Studies in elderly patients, who are at increased risk of cancer, are limited and controversial. Data for VDZ were shown in the GEMINI trials, where the incidence of malignancies was similar in all age subgroups [95].

### 4.4. Ustekinumab

A pooled analyses of six phase 2/3 studies for CD and psoriasis showed a good security profile for ustekinumab (UST) [83]. The incidence of malignancy (excluding NMSC) was low and comparable with that of the placebo (0.4 vs. 0.2 per 100 person-years). Combined across indications, the IRs for malignancies (excluding cervical cancer in situ and NMSC) in the UST and placebo groups were 0.6 (95% CI, 0.3–1.0) and 0.3 (95% CI, 0.0–1.9), respectively, with overlapping 95% CIs. UST has also not been associated with an increased risk of skin cancer [83].

Safety data of UST in elderly patients are limited. A real-world study from the ENEIDA registry shows that UST is also effective in elderly patients with CD compared to non-elderly population, and a higher rate of de novo neoplasms was observed, probably related to the older age [84]. Moreover, in the IM-UNITI trial, rates of malignancy were not increased between UST and placebo across all age subgroups [96,97].

### 4.5. Tofacitinib

Tofacitinib is an oral small molecule pan-Janus kinase (JAK) inhibitor, especially JAK 3 and JAK1, approved for the management of UC [98,99], whose efficacy and safety have been investigated in the OCTAVE trials (Oral Clinical Trials for tofacitinib in ulcerative colitis) [100].

This drug has been associated with an increased risk of neoplasms [100,101,102]. A higher frequency of NMSC in IBD patients receiving tofacitinib compared with the placebo was found; however, in four out of five cases, there was a history of NMSC, all patients had previous exposure to TPs and were in the 10 mg tofacitinib group. In the long-term extension (OLE) study, named OCTAVE Open, which included 944 IBD patients with follow-up of up to 7 years, the IRs for adjudicated NMSC were 0.96 (95% CI, 0.35–2.08), 0.68 (95% CI, 0.35–1.19) and 0.75 (95% CI, 0.45–1.19) for patients in the tofacitinib 5 mg, tofacitinib 10 mg and tofacitinib all groups, respectively, and the most common NMSC was basal cell carcinoma, with a total of 13 patients, mainly in the tofacitinib 10 mg group [103,104]. This increased risk of NMSC associated with tofacitinib has also been shown in rheumatoid arthritis (RA) and psoriasis [101,102].

Regarding malignancies other than NMSC, in the OCTAVE, open neoplasms were reported in 7 (4.0%) patients in the tofacitinib 5 mg group and 18 (2.3%) patients in the tofacitinib 10 mg group, with IRs of 1.03 (95% CI, 0.67–1.52), 1.09 (95% CI, 0.44–2.25), 1.00 (95% CI, 0.60–1.59) and 1.03 (95% CI, 0.67–1.52) for patients in the tofacitinib 5 mg, tofacitinib 10 mg and tofacitinib all groups, respectively [103,104]. Cancers described were four CRC, three breast cancer, two cervical cancer, two cancer of the penis, two soft tissue sarcoma, two cholangiocarcinoma, two non-Hodgkin lymphoma, two melanoma and two lung cancer.

An evaluation of tofacitinib safety data from clinical trials, including 1157 patients over a 4.4-year period (1612.8 patient-years of exposure), was also published [105]. The incidence rate of malignancies (excluding NMSC) was 0.7 (95% CI, 0.3–1.2). More than 80% of patients had been previously treated with anti-TNFs, and all of them were previously exposed to TPs. In the ORAL surveillance study in patients with rheumatoid arthritis treated with tofacitinib, compared with those receiving anti-TNFs, there was an increased risk of malignancies in a 5.5-year follow-up (6.1% for tofacitinib vs. 3.8% for anti-TNF, with an HR for adjudicated cancers—excluding NMSC—of 1.48; 95% CI 1.04–2.09). Age above 65 years and smoking were also risk factors [106].

There are not many real-world data in clinical practice on the safety profile of tofacitinib in UC patients, but in the study of Deepak et al., including 260 UC patients, there were only two malignancies [107], and in another real-world cohort of 113 IBD patients, only one patient developed a neoplasm, with previous immunosuppressive exposure [107,108].

In conclusion, there appears to be a risk of malignancies with the use of tofacitinib, which has been observed especially in some populations with RA. However, we need more long-term studies to define the risk of tofacitinib in IBD. Regulatory agencies consider it possible to estimate a similar (class) risk in other JAK inhibitors, such as filgotinib or upadacitinib; however, this has not yet been confirmed.

## 5. Treatments Decisions in Patients with IBD and Current or Previous Cancer

According to estimates from the World Health Organization (WHO) in 2019, the burden of cancer incidence and mortality is growing worldwide, to the point that cancer constitutes the first leading cause of death before the age of 70 years in many countries, including Europe and North America [109].

IBD therapy may impact the course of malignancies, and when a neoplasm appears, the focus of attention for the physician turns towards the treatment of cancer, with the interruption or not of IBD therapy being one of the most important dilemmas. Most clinicians tend to discontinue immunosuppressants and indicate more surgery [7,110]; however, maintaining IBD therapy while treating cancer could also be an option [7,111]. The reason why cessation is the most common trend is that some therapies, such as azathioprine, methotrexate or anti-TNF, can negatively impact the survival of some neoplasms such as CRC [112].

Discontinuation of therapy seems to be safe when the IBD is controlled. Axelrad et al. reported that 66.7% of patients diagnosed of an extra-intestinal malignancy during the course of IBD achieved remission during cytotoxic chemotherapy, while 17.4% developed active IBD, with a greater risk of reactivation in the group who received both chemotherapy and adjuvant hormone therapy (HR, 12.25; 95% CI, 1.51–99.06) [7]. Similar results were shown by the Massachusetts General Hospital, where only 17% of patients with an inactive IBD at the time of cancer diagnosis had a flare during a follow-up of 6 months, with those with a high risk of flare being younger patients with CD, those with prior history of anti-TNF therapy and those with previous hormonal therapy [111].

Related to radiotherapy in patients with IBD, gastrointestinal (GI) toxicity and 5-year survival seem to be similar between patients with and without IBD [110], so it seems to be a reasonable option for IBD patients.

**Figure 1:**
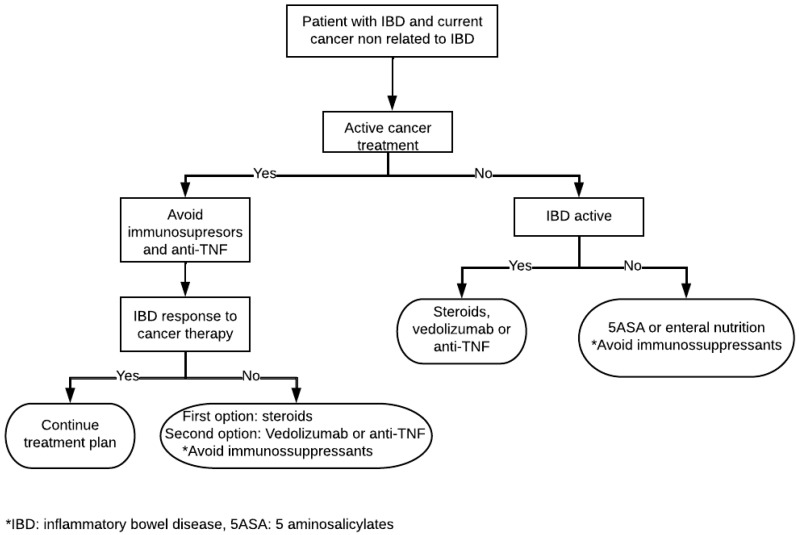
Practical management of patient with IBD and a current diagnosis of cancer.

In the case of the breakthrough of IBD activity, and after ensuring that symptoms are not due to chemotherapy or an infection, which are the more frequent causes of GI symptoms in IBD patients with active cancer, it is highly recommended to confirm the flare by endoscopy. When IBD flare is confirmed, consensus and expert opinion point to corticosteroids as the first and safest therapeutic option when IDB activity is uncontrolled; more selective biologic molecules, such as VDZ, if possible, or anti-TNFs, are preferable options as a backup plan in the absence of comparative studies. In patients suffering mild disease, 5ASAs and enteral nutrition are preferable [113]. A practical management approach for IBD patients with cancer is proposed in Figure 1.

## 6. Management of Patients with IBD and Previous Diagnosis of Cancer

In relation to IBD patients who have had cancer in the past, clinical guidelines often recommend restrictions in immunosuppressive and biological therapy. However, data are scarce, and most of the risks attributable to immunosuppressants come from studies in the post-transplant setting with a prior malignancy and high risk of recurrence: melanoma, NMSC, urinary cancer, lung cancer, renal cell cancer, breast cancer and myeloma, especially during the first 2 years post-malignancy [114].

In spite of that evidence, as previously exposed, some studies do not find an increased risk of neoplasms under immunosuppressive therapy [115]. For this reason, although NMSC seems to be the most common type of recurrent cancer, some experts suggest that TPs can be continued in this setting as these tumors are fully excised [116]. The situation is different in EBV-related lymphoproliferative disease, where TPs should not be used [117].

Related to anti-TNF therapy, some studies show that there is not a greater risk of developing new or recurrent cancer [115,118,119], but this excludes the risk of recurrent melanoma, a situation for which it seems better to avoid anti-TNFs [119]. Moreover, the ECCO consensus guideline recommends avoiding anti-TNF therapy during the first 2 years after cancer therapy cessation, or 5 for cancers with high risk of recurrence such as endometrial cancer, renal tract, melanoma, GI or lung cancer.

The experience with other biologic agents is limited regarding the risk of new cancer or recurrence in patients with IBD. As aforementioned, UST and VDZ seem to not be signals of increased malignancy risk [83]. Supporting this ascertain, Vedamurthy et al. demonstrated that the risk of cancer recurrence was similar in VDZ compared to anti-TNFs or no immunosuppressive regimens based on an IBD population with prior history of cancer [120]. However, as happens with UST, the follow-up period of these studies tends to be shorter (1 year) than in studies based on anti-TNFs. Moreover, it is important to highlight that most of the patients who developed a new or recurring cancer had been previously treated with other biologic agents, so no clear conclusions can be made.

**Figure 2:**
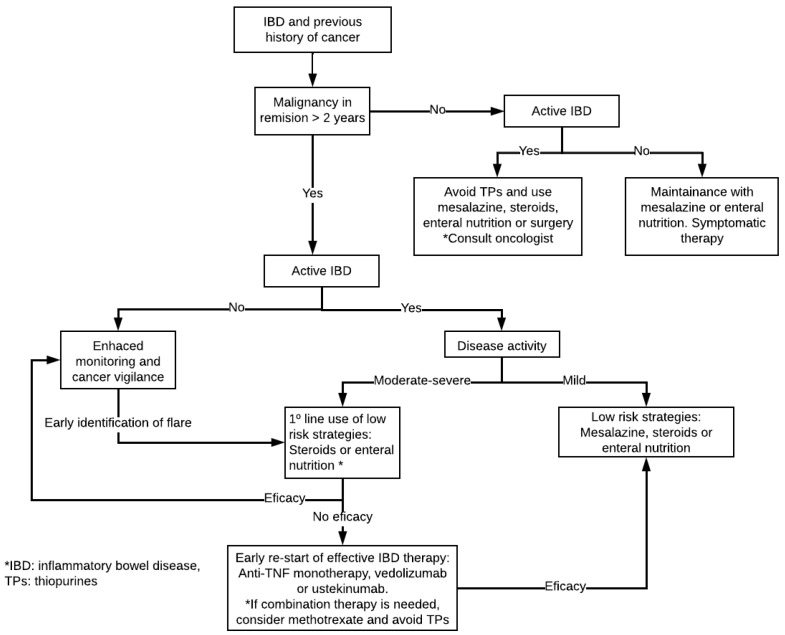
Suggested algorithm for the management of patients with IBD and prior history of cancer.

Finally, **tofacitinib** has been associated with an increased risk of cancer and cardiovascular disease when compared to anti-TNF drugs (HR, 1.48; 95% CI, 1.04 to 2.09), so it should not be used in patients at risk, as aforementioned, if possible [106]. An algorithm for the management of IBD patients with prior history of cancer is suggested in Figure 2.

## 7. Conclusions

The coexistence of IBD and cancer in the same patient makes the treatment of both pathologies a challenge. Discontinuation of therapy seems to be safe when IBD activity is controlled. However, in the case of relapse of inflammatory activity steroids, vedolizumab or anti-TNF is the most convenient therapeutic option, avoiding the prescription of immunosuppressants.

Although biologic use seems to be safe for patients with a current cancer or a prior history of cancer, as has been demonstrated in other chronic inflammatory conditions, we need to study specifically those patients with prospective studies to better answer how best to treat patients with a current or previous cancer. At the present, the I-CARE is an ongoing multicenter study, including 10,206 European IBD patients and aiming to investigate the risk of malignancy associated with IBD-therapy, especially biologic therapy [121]. Until long-term safety results are obtained from this large cohort, it is crucial to manage such conditions via the combined clinical expertise of the gastroenterologist and oncologist.

## Figures and Tables

**Table 1:** Colonoscopy surveillance recommendations according to the main international guidelines.

Risk	Characteristics	Next Surveillance Colonoscopy		
		ECCO 2017 [27]	ACG 2019 [30]	BSG 2019 [31]
**High**	-Stricture or dysplasia detected within the past 5 years -PSC (start screening at the time of diagnosis) -Extensive colitis with severe active inflammation -Family history of CRC in first-degree relative diagnosed younger than 50 years	1 year	PSC 1 year	1 year And extensive colitis with moderate activity
**Intermediate**	-Extensive colitis with mild active inflammation -Post-inflammatory polyps -Family history of CRC in first-degree relative diagnosed at 50 years and above	2–3 years And extensive colitis with moderate activity	1–3 years based on the combined risk factors for CRC and the findings of previous endoscopies	3 years
**Low**	No features of intermediate and high risk	5 years		5 years And left sided colitis and CD colitis affecting <50% of colon
**Pouch surveillance**	Risk factors: dysplasia or cancer identified after or at surgery, PSC, family history of CRC, severe pouchitis rapidly after pouch formation with moderate to severe villous atrophy, long retained rectal cuff	Risk factors: 1 year Asymptomatic patients: no surveillance		Risk factors: 1 year Asymptomatic patients: no surveillance or 5 years

ACG, American College of Gastroenterology; BSG, British Society of Gastroenterology; CRC, Colorectal Cancer; ECCO, European Crohn´s and Colitis Organisation; PSC, Primary Sclerosing Cholangitis.
